# Surgical management outcome and its associated factors among intestinal obstruction patients admitted to adult surgical ward of Wollega University Referral Hospital, Ethiopia

**DOI:** 10.1186/s12893-023-02043-1

**Published:** 2023-05-17

**Authors:** Adisu Tafari Shama, Olana Terefa, Iyasu Gadisa, Gemechu Feyera, Getnet Tamiru, Dufera Rikitu Terefa, Emiru Merdassa

**Affiliations:** 1 Department of public health, Institute of health sciences, Wollega University, Nekemte, Ethiopia; 2 School of Medicine, Institute of health sciences, Wollega University, Nekemte, Ethiopia

**Keywords:** Intestinal obstruction, Management outcome, Surgical management, Surgical management outcome, Management outcome for intestinal obstruction, Associated factors, Nekemte, Ethiopia

## Abstract

**Background:**

Globally, bowel obstruction is the most common cause of surgical emergencies. It remains a challenge to healthcare workers in spite of improvements in management techniques. There is a lack of the study to determine the surgical management outcome and its associated factors in the area of study. Hence, this study aimed to determine management outcome and its associated factors among surgically treated intestinal obstruction patients at Wollega University Referral Hospital, 2021.

**Methods:**

Facility-based cross-sectional study was conducted among all cases surgically managed for intestinal obstruction between September 1, 2018 and September 1, 2021. Data were collected using a structured checklist. The collected data were checked for completeness and entered into data entry software and then exported to SPSS version 24 for data cleaning and analysis. Both bi-variable and multivariable logistic regressions were run. P-value < 0.05 was used to declare a statistically significant association in multivariable logistic regression. The odds ratio along with 95%CI was estimated to measure the strength of the association.

**Result:**

116 (59.2%) patients had favorable surgical management outcome for intestinal obstruction. Male sex (AOR = 3.694;95%CI:1.501,9.089), absence of fever (AOR = 2.636; 95%CI:1.124,6.18), ≤ 48 h duration of illness before operation (AOR = 3.045; 95%CI:1.399,6.629), viable intraoperative bowel condition (AOR = 2.372; 95%CI:1.088, 5.175), having bowel resection and anastomosis (AOR = 0.234; 95%CI:0.101,0.544) were the significantly associated factors of the favorable surgical management outcome for intestinal obstruction.

**Conclusion and recommendation:**

The favorable management outcome of patients with intestinal obstruction who were treated surgically in this study was low. Factors like sex, fever, short duration of illness, viable intraoperative bowel condition, and bowel resection and anastomosis were found to influence the surgical management outcome of patients with intestinal obstruction. The patient with intestinal obstruction should seek health care on time. Health professionals have to be skilled and provide appropriate care for the patients to reduce the risk of complications.

## Background

Intestinal obstruction (IO) is defined as the interrupted or impaired passage of intestinal contents [1]. Based on its anatomical location, obstruction can involve; the small intestine, large intestine, or both the small and large intestines (generalized ileus). More than three-quarters 76% of IO occurs in the small intestine [2]. Being the leading cause of acute abdomen, it is a global problem that is consuming many resources in terms of surgical services, especially for countries found in the ‘volvulus belt’ regions including Africa, India, Iran, Russia, and Brazil [3]. It is also a challenge in causing mortality which ranges from 3 to 30% all over the world [4, 5]. Adhesions, neoplasms, and herniation are the most common causes of acute intestinal obstruction [6].

There are different treatment options for IO depending on the cause and extent of the obstruction. Some of them require surgical opening of the abdomen while others do not require an operation. A low-fiber diet, enema, tube deflation, decompression, and self-expanding metal stents are among the treatments [7]. Surgical intervention either minimally invasive laparoscopic surgery or complicated open surgical procedure is also used to treat intestinal obstruction [8].

Annually, millions of people undertake surgical management which accounts for an estimated 13% of the world’s total disability-adjusted life years, 0.5-5% crude mortality rate, and 25% postoperative complications [3, 9]. In Ethiopia, the prevalence of unfavorable surgical management outcome for intestinal obstruction ranges from 13.6 to 26.5% [5, 10–18]. Besides, unfavorable surgical management outcome is more likely in operation for gangrenous large bowel obstruction (LBO) than SBO [17].

For the appropriate management, the world society of emergency surgery updated the Bologna guidelines for the diagnosis and management of adhesive small bowel obstruction in 2017 [19]. There are advancements in the field of medicine, the introduction of a safe surgery checklist, improved monitoring, and related safety practices during anesthesia, surgical technique, and conservative management. However, the surgical management outcome of intestinal obstruction remains a challenge to the healthcare system [20]. Regardless of its underlying causes, surgery for IO sometimes led to a variety of post-operative complications such as incision site infections, wound dehiscence, pneumonia, and sepsis which are not rare, especially after emergency surgery for IO and even death as the poor outcome of that surgical management [6].

The incidence of intestinal obstruction and its surgical management outcome may be affected by different factors. This can be patient-related and clinical-related factors. Some of these factors include the cause of obstruction, age, poor health-seeking behavior, duration of illness before surgery, length of hospital stays after surgery and comorbidity, presence of peritonitis, hematocrit level, and complication detection time which are strongly associated with morbidity and mortality [16, 21–25].

Even though, the studies were conducted in other parts of Ethiopia, no prior study was conducted in the study area regarding the surgical management outcome of intestinal obstruction patients and its associated factors. The result of this study could help clinical practitioners to evaluate the quality of their surgical procedures and work to improve the service quality. Furthermore, this study might contribute to the improvement of service quality by identifying the factors affecting surgical management outcome and then will benefit the patients. For the policymakers and program planners, these data provide epidemiological and clinical information that will serve as essential input to design proper strategies to address IO. Thus, this study was conducted to generate baseline information about the management outcome of IO and its associated factors among surgically managed intestinal obstruction patients in Wollega University referral hospital.

## Methods and materials

### Study area

The study was conducted at Wollega University Referral Hospital (WURH) which is located in Nekemte town, western Ethiopia. Nekemte town is located 331 km far away from Addis Ababa the capital city of Ethiopia. Wollega University Referral Hospital was established in 2016 and functions as a practical training center for more than fourteen departments and provides prevention and curative services for over five million catchment populations. Among the 279 Hospital beds, 42 of them are found in the surgical ward. Minor, elective, and emergency surgical procedures are given in the surgery department including outpatient services. There were nine (9) surgeons in the hospital who were permanent employees of the hospital. Among these, seven (7) of them were general surgeons while 2 were orthopedic surgeons. Additionally, there were 22 surgical residents in the hospital. All of them provide surgical services except year 1 and 2 residents. Between September 1, 2018 to September 1, 2021, 1872 patients were admitted to the surgical ward of the WURH. Of these, 523 of them were admitted with the diagnosis of acute abdomen from which, 206 were admitted with IOs and received surgical intervention.

#### Study design and study period

A retrospective chart review was done from September 27, 2021 to October 8, 2021.

### Population

#### Source population

The source populations were all cases surgically managed for intestinal obstruction at WURH from September 1, 2018 to September 1, 2021.

#### Study population

The study population includes all cases that fulfilled the inclusion criteria from the cases surgically managed for intestinal obstruction at WURH from September 1, 2018 to September 1, 2021.

### Inclusion and exclusion criteria

#### Inclusion criteria

The study inclusion criteria were admission with the diagnosis of IO and treatment in the surgical ward of the Wollega University referral Hospital from September 1, 2018 to September 1, 2021.

#### Exclusion criteria

Patients who had incomplete records (i.e., missing important information on causes and management outcome-related variables) and patients whose records were completely lost were excluded from the study [15]. Accordingly, 10 (5%) patient charts were excluded being incomplete.

### Sample size determination

The census of all the 206 patients managed for the IO at WURH was intended while 196 had satisfied the inclusion criteria and were included.

### Data collection tools and method

#### Data collection instrument, Data collection procedures and data collectors

The data were collected by reviewing the registration books, anesthesia charts and patient charts using structured checklists that were prepared by principal investigators after reviewing different literature. A checklist was developed in the English language to collect important information such as age, sex, admission diagnosis, intraoperative findings, intra-operative procedures, duration of presentation, causes of IO, postoperative complications and management outcome. First, the medical registration numbers of patients in the study period were identified from registration books (logbooks), and then their charts were retrieved from the card office. Then, information from patient cards was extracted into the structured format [15]. Respectively, two (2) diploma holders and one first-degree holder clinical nurses collected and supervised the data.

### Study variables

#### Dependent variable

Management outcome.

#### Independent variables

Socio-demographic characteristics such as age, sex, residence, and occupation; pre-operative clinical characteristics like presenting symptoms, duration of illness, preoperative diagnosis, preoperative care received, co-morbidity, and previous abdominal surgery; intra and postoperative clinical characteristics, intraoperative diagnosis, type of intraoperative surgical procedure done, and postoperative antibiotics received were the independent study variables.

### Operational definitions

#### Intestinal obstruction

Intestinal obstruction is the prevention of passage of intestinal contents [26]. The information was abstracted from the patient chart based on the diagnosis made by the physician. The assigned physicians diagnosed intestinal obstruction based on a combination of diagnostic options including clinical diagnosis (history, and physical examination), and imaging (plain abdominal X- ray, and ultrasound).

#### Surgical management

managing patients with IO with surgical exploration or operations performed on the abdomen to relieve the causes of obstruction [26].

#### Surgical management outcome

is the condition of the patient after the surgical procedure is done. First, each post-operative condition of the patient was assessed and reported separately whether it was death, other complications, or comfortable. Then the post-operative conditions were then categorized into two main categories and analyzed. Accordingly, the patient was categorized as having unfavorable surgical management outcome either if died or developed any postoperative complications (including wound infection, facial dehiscence, anastomotic leakage, developed septic shock, pelvic collection, and pneumonia) until he/she was discharged from the hospital. In contrast to this, the patient was categorized as having favorable surgical management outcome if he/she developed neither postoperative complication nor death after conservative or operative management for IO during the stay in the hospital [10–17].

### Data quality control

Before the data collection period, the checklist was pretested on 5% of the sample size in a non-selected health facility (Nekemte specialized hospital) to identify any ambiguity, inconsistency, and acceptability of the checklist. The training was given to the data collectors and supervisor on the data collection procedures, the purpose of the study, and ethical issues by the investigators. To avoid interpersonal variation between data collectors, the two data collectors were retained throughout the data collection process. Regular daily supervision by the supervisor was done for the consistency and completeness of checklists. The completed checklists were checked for completeness and consistency at every step of data collection. After data collection and before starting data analysis, checklist completeness was rechecked.

### Data processing, and analysis

The collected data was checked for its completeness, coded, and entered into Epidata version 3.1 (the EpiDataAssociation, Odense Denmark), then exported to statistical package for social sciences (SPSS) version 24.0 (IBM, Armonk, New York, U.S.) for data cleaning and further descriptive, and inferential analysis. Results were presented using frequency tables, graphs, and percentages. Purposeful variable selection was done to build the multivariable model by considering both the statistical and clinical significance of independent categorical variables. Bivariable logistic regression analysis was done to determine the associations between independent variables and dependent variable. Variables with a p-value of less than 0.25 in the bivariable binary logistic regression analysis were entered into multivariable logistic regression. Model fitness was checked using Hosmer and Lemeshow’s test and it was found to be insignificant (p-value > 0.05) [27]. Multicollinearity was checked using variance inflation factor (VIF) and no collinearity problem was diagnosed having VIF > 10. Multivariable binary logistic regression was run to identify the independent factors of the management outcome of IO. Adjusted odds ratio with 95% confidence interval and p-value < 0.05 were used to declare statistically significant association in multivariable logistic regression.

## Result

### Socio-demographic characteristics

From the intended sample size of 206, data from 196 (95%) patients were retrieved for further analysis. The mean age of the patients was 42.08 ± 14.6 years with the minimum and maximum ages of 15 and 80 years respectively. The study revealed that 137 (69.9%), 151 (77.0%), and 126 (64.3%) of the study participants were males, from rural areas, and farmers respectively (Table 1).

**Table 1:** Socio-demographic characteristics of the surgically managed intestinal obstruction patients from 2018 to 2021 (N = 196)

Variables		Frequency (number with %)
Age in years	15–24	17 (8.7%)
	25–34	44 (22.4%)
	35–44	47 (24.0%)
	45–54	37 (18.9%)
	55–64	33 (16.8%)
	>= 65	18 (9.2%)
Sex	Male	137 (69.9%)
	Female	59 (30.1%)
Residence	Rural	151 (77.0%)
	Urban	45 (23.0%)
Occupation	Farmer	126 (64.3%)
	Government employee	21 (10.7%)
	Self-employed	24 (12.2%)
	Student	13 (6.6%)
	Others^a^	12 (6.1%)

^a^ Daily laborer, house wife

### Preoperative history, clinical presentation, and diagnosis

All 196 (100%) of the intestinal obstructions were due to mechanical obstruction. All of the patients 196 (100.0%) reported abdominal pain, nausea and vomiting 196 (100.0%) whereas nearly three quarters 153 (78.1%) versus 141 (71.9%) reported failure to pass feces, and failure to pass both feces and flatus, respectively. In addition, two-thirds 131 (66.8%) had abdominal distension while about a quarter 44 (22.5%) were presented with fever.

The majority of the cases 132 (67.3%) came 48 h after their illness started and 27 (13.8%) of all IO cases had at least one diagnosed co-morbid disease like cardiac disease, renal disease, and hypertension. This study also showed that 27 (13.8%) had a previous history of abdominal or pelvic surgery. Depending on the bowel involvement, nearly more than half 102 (52%) were diagnosed with small bowel obstruction (Table 2).

**Table 2:** Preoperative history, clinical presentation, and diagnosis of patients managed for IO in WURH, from 2018 to 2021 (N = 196)

Variables		Frequency ( %)
Clinical presentation	Abdominal pain	196 (100.0%)
	Nausea and Vomiting	196 (100.0%)
	Failure to pass feces	153 (78.1%)
	Failure to pass feces and flatus	131 (71.9%)
	Abdominal distension	141 (66.8%)
	Fever	4 (2.0%)
	abdominal swelling	102 (52.0%)
Duration of the illness	<=48 h	64 (32.7%)
	> 48 h	132 (67.3%)
Had chronic illness	Yes	27 (13.8%)
	No	169 (86.2%)
Types of chronic illness (n = 27)	Hypertension disorders	19 (9.7%)
	Cardiac disease	6 (3.1%)
	Renal disease	2 (1.0%)
Had previous abdominal or pelvic operation (n = 196)	Yes	27 (13.8%)
	No	169 (86.2%)
Reason for the operation done (N = 27)	Appendicitis	4 (2.0%)
	Peritonitis	4 (2.0%)
	Gynecologic and obstetric cases	3 (1.5%)
	Unknow	10 (5.1%)
	Others b	6 (3.1%)
Kinds of IO depending on bowel involvement	SBO	102 (52.0%)
	Large bowel obstruction (LBO)	94 (48.0%)

^b^A ganglionic colon, abdominal injury, hernia repair

### Peri-operative care, Procedure done, intra- and postoperative clinical characteristics

Concerning the key elements of preoperative care assessed in this study, intravenous fluid resuscitation was given and a Naso-gastric tube (NGT) was inserted for all (100.0%) patients; rectal tube deflation was done for 46 (23.5%) patients, and preoperative prophylactic antibiotics were given for 189 (96.4%) patients. The most surgical procedure done was resection and anastomosis 142 (72.2%) followed by derotation 25 (12.8%) and band release 24 (12.2%). In this study, most small bowel obstruction was found to be secondary to viable small bowel volvulus 31 (30.4%) followed by adhesion 23 (22.5%). The most common cause of large bowel obstruction was viable sigmoid volvulus 40 (42.5%) followed by colorectal cancer 25 (26.6%). Regarding the length of hospital stay, 116 (59.2%) of patients stayed in the hospital for < 8 days (Table 3).

**Table 3:** Peri-operative care given, procedure done, intra- and postoperative clinical characteristics of patients managed for IO in WURH from 2018 to 2021 (N = 196)

Variables		Frequency (%)
Perioperative care given	NGT decompression and fluid resuscitation	196 (100.0%)
	Rectal tube deflation and fluid resuscitation	46 (23.5%)
Antibiotics given	Yes	189 (96.4%)
	No	7 (3.6%)
Types of antibiotics given (n = 189)	Ceftriaxone only	71 (36.2%)
	Ceftriaxone and metronidazole	120 (61.2%)
	Others ^c^	5 (2.6%)
Types of intraoperative surgical procedure done	Resection and Anastomosis	142 (72.4%)
	Derotation	25 (12.8%
	Adhesiolysis and band release	24 (12.2%)
	Manual reduction	5 (2.6)
Cause of SBO and Intra operative findings (n = 102)	Viable Small bowel volvulus	31 (30.4%)
	Adhesion	23 (22.5%)
	Intussusceptions	14 (13.7%)
	Hernia	8 (7.8%)
	Gangrenous small bowel volvulus	8 (7.8%)
	Ilio-sigmoid knotting	8 (7.8%)
	Others^d^	8 (7.8%)
Cause of LBO and Intra operative finding (94)	Viable Sigmoid volvulus	40 (42.5%)
	colorectal ca.	25 (26.6%)
	Gangrenous sigmoid volvulus	20 (21.3%)
	Ilio-sigmoid knotting	4 (4.2%)
	Intussusceptions	1 (1.1%)
	Others ^e^	4 (4.2%)
Length of hospital stay	<=8days	116 (59.2%)
	> 8days	80 (40.8%)

^d^ neoplasms, ^e^ Cecal mass, mesenteric ischemia, fecal impaction, Gastro-intestinal stromal tumor/GIST

### Surgical management outcome

This study showed that 9 (4.6%) of the surgically managed patients died. The majority 178 (90.4%) of the patients including those who faced postoperative complications were improved and discharged while the rest 9 (4.6%) were categorized as others (leave against medical advice/LAMA, referred for chemo/radiotherapy, status not documented, and unknown).

**Fig. 1:**
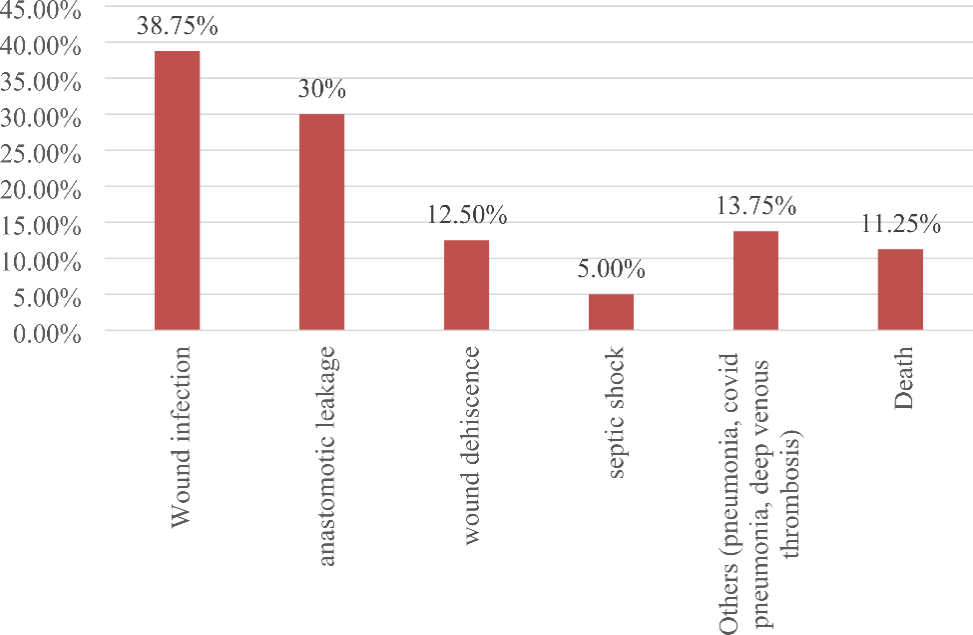
Types of post-operative complications among patients who had unfavorable surgical management outcome in Wollega University referral hospital from 2018–2021 (n = 80)

The most common unfavorable complication that followed the surgical management was wound site infection 31 (38.7%) followed by anastomotic leakage 24 (30.0%) (Fig. 1). Overall, 116 (59.2%) of patients in this study had favorable surgical management outcome whereas 80 (40.8%) had unfavorable surgical management outcome.

#### Factors associated with management outcome of IO

In the bivariable logistic regression, sex, occupation, residence, having bowel resection & anastomosis, intraoperative bowel condition, illness duration, fever, and kinds of IO were significantly associated with management outcome. Nevertheless, only five factors showed significant association with management outcome in multivariable analysis. Accordingly, males were found to have 3.69 more odds of favorable intestinal obstruction management outcome than females. The odds of favorable management outcome for the patients who had a bowel resection and anastomosis was 77% (0.234; 95%CI: 0.101, 0.544) less likely as compared to their counterparts. Surgically managed patients with viable intraoperative bowel conditions had favorable outcome 2 times more likely than the patients with a gangrenous bowel condition. The patients who had the illness for less than or equal to 48 h prior to surgical management had favorable management outcome 3 times more likely as compared to those having an illness of > 48 h. Those patients presented with no fever had 2.63 times more odds of favorable management outcome than their counterparts (Table 4).

**Table 4:** Factors associated with management outcome of surgically managed IO patients in WURH (n = 196)

Variables	Category	Management outcome		COR with 95%CI	P-value	AOR with 95%CI	P-value
		Favorable n (%)	Unfavorable n (%)				
Having dowel resection & anastomosis	Yes	100(70.4%)	42 (29.6)	0.177(0.089,0.351)	0.000*	0.234(0.101,0.544)	0.001**
	No	16 (29.6)	38 (70.4)	Ref	Ref	Ref	ref
Intraoperative bowel condition	Viable	95(65.5%)	50(34.5%)	2.714(1.411–5.222	0.003*	2.372(1.088–5.175)	0.030**
	Gangrenous	21(41.2%)	30(58.8%)	Ref	Ref	Ref	Ref
Duration of illness	<=48 h	63(71.6%)	25(28.4%)	2.615(1.439–4.753)	0.002*	3.045(1.399–6.629)	0.005**
	> 48 h	53(49.1%)	55(50.9%)	Ref	Ref	Ref	ref
Fever	Yes	20(42.6%)	27(57.4%)	Ref	Ref	Ref	Ref
	No	96(64.4%)	53(35.6%)	2.445(1.253,4.771)	0.009*	2.636 (1.124–6.18)	0.026**
Kinds of IO	SBO	49(48.0%)	53(52.0%)	2.684(1.485,4.852)	0.001*	(1.157,0.5232.562)	0.719
	LBO	67(71.3%)	27(28.7%)	Ref	Ref	Ref	Ref
Antibiotics given	Yes	113(59.8%)	76(40.2%)	Ref	ref	Ref	ref
	No	3(42.9%)	4(57.1%)	1.982(0.431,9.109)	0.379		
Types of antibiotics given	ceftraxone + metrindazole	74(61.7%)	46(38.3%)	Ref	ref	Ref	ref
	ceftraxone only	39(54.9%)	32(45.1%)	1.320(0.728,2.393)	0.360		
	Not given	3(60.0%)	2(40.0%)	1.072(0.173,6.663)	0.940		
Previous abdominal or pelvic operation	Yes	16(59.3%)	11(40.7%)	0.996(0.436,2.277)	0.993		
	No	100(59.2%)	69(40.8%)	Ref	ref		
The patient had Chronic illness	Yes	18(66.7%)	9(33.3%)	0.690(0.2931.625)	0.396		
	No	98(58.0%)	71(42.0%)	Ref	ref		
Occupation of the patient	Farmer	66(52.4%)	60(47.6%)	Ref	ref	Ref	ref
	Government employ	17(81.0%)	4(19.0%)	0.259(0.082.812)	0.021*	0.194(0.021–1.791)	0.148
	Self employed	19(79.2%)	5(20.8%)	0.289(0.102.823)	0.020*	0.232(0.035–1.543)	0.131
	Student	6(46.2%)	7(53.8%)	1.283(0.4084.033)	0.669	1.272(0.147–11.038)	0.827
	Others	8(66.7%)	4(33.3%)	0.550(0.1581.920)	0.349	0.790(0.101–6.201)	0.822
Residence	Rural	85 (56.3%)	66(43.7%)	1.719(0.847,3.491)	0.134*	0.587(0.085–4.074)	0.590
	Urban	31(68.9%)	14(31.1%)	Ref	ref	Ref	Ref
Sex of the patient	Male	73(53.3%)	64(46.7%)	2.356(1.2124.581)	0.012*	3.694(1.501–9.089)	0.004**
	Female	43(72.9%)	16(27.1%)	Ref	ref	Ref	Ref

* Significant at p-value < 0.25, **significant at p-value < 0.05, COR-Crude Odds Ratio, AOR: Adjusted Odds Ratio, CI-confidence interval, ref-refence category

## Discussion

This study found that 116 (59.2%) of patients managed for bowel obstruction had favorable management outcome. Although this result is comparable to the result of the study in Turkey (58.1%) [22], it is lower than the findings of the study done in Nekemte specialized hospital 73.5% [10], Adama Hospital 75.4% [11], Asella hospital-75.7% [12], Arba Minch General Hospital-77.7% [13], Chiro Hospital-78.7% [14], Dilla Hospital-86.4% [15], University of Gondar Comprehensive Specialized Hospital-83.3% [16], Debre Birhan Referral Hospital-83.3% [5], 82% in south Wollo Zone Hospitals [17], 77% in Wolayita [18], Kenya-86.4% [28], and India-74% [29]. The possible reason for the observed discrepancy might be due to the difference in the professionals involved in the surgical management, the difference in the study settings, study period, and population. The relatively low favorable outcome revealed in this study might be attributed to the impact of coronavirus disease (COVID-19) on health care as the period covered in this study was the period of the COVID-19 outbreak. Particularly, health workers in the Wollega University referral hospital were overburdened by COVID-19-related activities as the hospital was serving the COVID-19 treatment center.

The most postoperative complications were wound site infection 31 (38.75%) followed by anastomotic leak 24 (30%), wound dehiscence 10 (12.5%), septic shock 4 (5%), and others (like pneumonia, deep venous thrombosis, COVID pneumonia) 11 (13.75%). This condition is almost similar to the studies done at Nekemte, Dilla and Adama Hospitals which showed that the most common postoperative complication was wound site infection [10, 11, 15].

The overall mortality rate in this study was 9 (4.6%) among a total of 196 analyzed cases who were engaged for surgical management while it was about 11.25% among those patients who developed complications. Lastly, the majority 178 (90.4%) of the patients who underwent surgical management were improved and discharged. This is in line with a study done at Dilla University which showed an overall mortality rate of 4.7% [15] and Asella Hospital 4.5% [12] but greater than the finding of the study done at Adama Hospital which showed the overall mortality rate of 2.5% [11] while less than the one from India 7.98% [29]. Regarding the relationship between death and the cause of SBO, the proportion died was almost similar in both cancer-caused (12.5%) and non-cancer-caused (12.3%) SBO. However, 16% of patients with LBO due to colon cancer died whereas only 7.7% of LBO due to non-cancer causes died in this study.

Even though not statistically significant, the majority (59.4% of those patients who received the antibiotics had favorable surgical management outcome than those patients who didn’t receive the antibiotics (42.9%). This could be due to the fact that antibiotics that are given as either prophylaxis or treatment [30] might have a crucial role in fighting infections and then reduce unfavorable surgical management outcome including mortality.

Those surgical patients to whom bowel resection and anastomosis procedures were performed were less likely to have favorable management outcome as compared to their counterparts. Although this was in contradiction with the study done in Dilla Hospital [15], it is in line with the study done at Adama Hospital [11], and Kenya [28] in which resection and anastomosis have significantly increased the outcome of unfavorable management outcome.

In our study, favorable management outcome was affected by gangrenous intraoperative finding. This is supported by the study conducted in Wolayita [18], Asella Hospital [12], Arba Minch General Hospital [13], Chiro, Dilla, and Adama Hospitals in which patients with viable small bowel volvulus and viable sigmoid volvulus were more likely to have favorable surgical management outcome compared to their counterparts [11, 14, 15].

Duration of illness before the surgical intervention was one of the factors that influence favorable management outcome in this study. This is in line with the study done in Nekemte specialized Hospital [10], Adama Hospital [11], Rwanda [31], Gondar [16], Dilla [15], Gurage [26], Wolayita [18] and study done at Chiro Hospital [14] that showed patients who came late and delayed the surgical intervention had more probability to develop unfavorable management outcome. This might be attributed to the rapid progression of obstruction effect to other neighbor organs that could lead to poor management outcome as time goes on. Besides, delay in health care seeking could lead to delay in diagnosis and treatment that could lead to unfavorable management outcome including death.

In our study, patients who had no fever were more likely to have favorable management outcome when compared with patients who had a fever. This is against with study done at Dilla Hospital in which those patients who were having fever were less likely to develop an unfavorable outcome [15]. The possible reason for the positive association between the absence of fever and favorable management outcome might be that fever is due to underlying infections and other complications which could contribute to poor management outcome.

The odds of favorable management outcome for males were 3.69 times more likely than for females. A similar finding was reported in the previous study conducted in Nekemte specialized hospital [10]. The possible explanation for the observed association in the current study might be that majority of participants in this study were males (2.3 to 1 ratio). Almost similar findings have been reported in other studies done in Tanzania [32] and Dilla University Hospital [15] which showed most of the participants were males.

This study is not without limitations. The first limitation is that some of the essential parameters like the educational status of the patients and household income which may significantly contribute to the unfavorable outcome of IO were not measured in this study since secondary data were utilized. Secondly, the cross-sectional nature of the study doesn’t give confidence to certainly describe the causal effect of the associated factors. Lastly, there might be a misdiagnosis regarding intestinal obstruction as the authors just used the diagnosis from the patient chart.

## Conclusion and recommendation

The 59.2% favorable surgical management outcome in this study was found to be low as compared to the studies conducted in other parts of Ethiopia. However, the majority of the surgically managed intestinal obstruction patients and those who developed complications after surgical intervention were improved and discharged. Sex, absence of fever, short duration of illness before surgery, viable intraoperative bowel condition, and not undergoing bowel resection and anastomosis were the factors that were significantly associated with the favorable surgical management outcome of intestinal obstruction.

It would be better if the patients with intestinal obstruction seek health care on time to avoid and/or minimize the complications. Health workers should also manage the patient timely with appropriate treatments as per the guideline. On this regard, availing skilled personnel would be helpful to manage the patients appropriately that would lead to have favorable surgical management outcome for the intestinal obstruction and reduce the risk of post-operative complications.

## Data Availability

The datasets analyzed during the current study are available from the corresponding author upon reasonable request.

## Abbreviations

COVID: Corona virus disease
IO: Intestinal Obstruction
LBO: Large Bowel Obstruction
NGT: Naso-gastric Tube
SBO: Small Bowel Obstruction
WURH: Wollega University Referral Hospital
